# Racial Differences Affecting Night Time Blood Pressure Dipping Groups in Hypertensive Patients

**Published:** 2016-02-29

**Authors:** LH Wong, Huang Elaine, RT Kong

**Affiliations:** 1 College of Medicine and Health, University College Cork, Ireland; 2 School of Medicine and Medical Science, University College Dublin, Ireland

**Keywords:** Blood pressure, Nocturnal dip, Hypertension, Racial difference, White, Asians

## Abstract

**Background:**

Normal blood pressure (BP) follows a circadian rhythm, with dipping of BP at night. However, little has been done to show how the dipping groups vary amongst the White and Asian population at different periods of the year. This study aims to examine the pattern of nocturnal dipping between the White and Asian population, as well as to compare it to the different timings of the year, between summer and winter.

**Methods:**

Ambulatory Blood Pressure Monitor recordings were obtained from 220 patients, half were White patients obtained from Mercy University Hospital, Cork, Ireland and half were Asian patients from National Heart Centre, Singapore during the summer period from May to June and the winter period from October to December.

**Results:**

Both the Irish and Singaporeans exhibit a decrease in total number of reverse dipper from summer to winter. However, the redistribution of reverse dipper was mainly to the dippers in Singapore, while in Ireland it was to both the extreme dipper and dipper.

Irish seasonal changes also resulted in an increase in nocturnal diastolic pressure (95% CI, 0.72 to 6.03, 3.37 mm Hg; p<0.05) and a change in the duration of dipping at night (95% CI, 0.045 to 1.01, 0.53 Hours; p<0.05).

**Conclusion:**

Regardless of race or temperature, reverse dippers seem to decrease in winter. However, the racial differences dictate the redistribution of the fall in number of dippers. This has implications on how reverse dippers should be treated at different periods of the year.

## Introduction

Blood Pressure (BP) dipping is defined as the difference in BP from average waking to sleeping measurement. BP follows normal circadian rhythm of dipping at night at about 10-15% as compared to the day. Night time dipping is further divided into 4 types, reverse dipper (<0%), non-dipper (0-10%), dipper (10-20%) and extreme dipper (>20%). The dip is thought to be affected by an inability to excrete sodium during the daytime [1]. It has also been found to be greatly reduced in populations who are regularly exposed to seasonal changes [2].

Studies have shown how different dipping status of blood pressure during the night time affects the prognosis of diseases [3-5]. This is especially so in the case of reverse dippers, which are frequently linked to higher mortality in Cardiovascular Diseases (CVD), perhaps related to nocturnal hypoperfusion and/or an exaggerated morning BP surge [6-12].

This shows the importance in knowing the pattern of dipping in different races. With this knowledge, there should be an impact not only on the choice of the medications but also how clinicians should pay attention, not just to the reverse dippers but also other dippers during different seasons due to the tendency to convert to reverse dippers.

Lianne et al. have shown the differences in night time blood pressure dipping between the Black and White populations [13]. Other researchers such as Fumo et al. have compared between American blacks and whites, as well as African blacks and found a smaller reduction in mean blood pressure at night in American blacks compared to other groups [14]. Little however, has been done to show the relationships between Asian and White population.

This study aims to elucidate the racial difference of nocturnal dipping in patients with a clinical diagnosis, or suspicion of hypertension. This research also attempts to elicit the difference in dipping status between summer and winter.

By exploring the effect of racial differences on the types of nocturnal dip status, particularly between the White and Asian population, we will be able to provide clinicians with a better appreciation of the factors that influence the successful control of hypertension. Treatment would thus be more individualized, based on the races of each of the individuals.

## Methods

This is a retrospective cohort study where patients who were referred to Mercy University Hospital (MUH), Cork, Ireland and National Heart Centre (NHC), Singapore were considered for the study. Only patients who were purely hypertensive without any other comorbidity were included. Hypertensive patients that were selected for the study were randomly selected by a non-stratified method. Hypertensive patients refer to patients who have a systolic pressure greater than 140 mm Hg and diastolic pressure of 90 mm Hg on ABPM. Of which, people with the following conditions were excluded from the study: Type II Diabetes Mellitus; renal diseases such as renal stenosis and renal failure; endocrine disorders like Pheochromocytoma, Addison’s disease and Cushing’s disease.

The diagnosis is made according to National Institute for Health and Care Excellence (NICE) guidelines of hypertension medications in both countries [15]. Those that were shown to be taking any other forms of non-anti-hypertensive medications that might alter the BP were excluded from the study. Patients were continued to be monitored each visit every 6 months. Patients in Ireland from certain occupations such as farmers will be excluded as these occupations will not be found in Singapore.

The data being collected have been ethically approved by the Clinical Research Ethical Committee (CREC) of UCC and the Centralised Institutional Review Board (CIRB) of Singapore.

Data on Systolic blood pressure, diastolic blood pressure are recorded by 24 hours Ambulatory Blood Pressure Monitor (ABPM). Race, age, gender, Body Mass Index (BMI), any present diagnosis, family history, anti-hypertensive or diuretic drugs that affects blood were recorded from the Citrix system and medical reports in Singapore and Ireland. Confounding factors include: Smoking or chewing tobacco; alcohol consumption; recreational drugs; age differences brought about by arterial stiffness and calcification, are controlled as well; occupations of different stress level and the number of working hours per week as higher level of stress will inevitably result in an increase in blood pressure. Comorbidities include Hyperlipidaemia and Ischaemic heart disease.

The Ambulatory Blood Pressure Monitor used in Singapore was a Spacelabs Model 90217 while those used in Ireland were Meditech LTD ABPM-04, Medtech LTD ABPM-05 and Spacelabs model 90207. The MUH ABPM data were analysed using the DABL software system, DABL Health, Dublin, Ireland. The ABPM’s were programmed to record BP at thirty minutes intervals over the day time and the night time. Systolic and diastolic BP and heart rate (HR) values were averaged to each hour of the recording, over the daytime (from 08:00–20:00 hours), over the night time (from 01:00-06:00 hours), and over the entire 24-hour periods [16].

The retrospective data were collected from the period of May to July and October to December 2013. 110 patients’ details were collected from each period amounting to 220 patients per country.

Data are also expressed in terms of means with standard deviations using descriptive statistics or independent sample T test is used. The type of dippers will be assigned a number, for e.g. 1: Reverse dipper, 2: Non dipper, 3: Dipper and 4: Extreme dipper. Sexes and races will be keyed in through the same method as well. However, the duration of dipping and the values of systolic and diastolic pressure will be keyed in using the actual values.

Data on temperature, humidity and other metereological data was be obtained from the Irish Meteorological Service (MET) and from the National Environment Agency (NEA) of the Singapore government.

Data on the electricity consumption by household in Singapore was taken from the Energy Market Authority (EMA) of Singapore.

## Results

### Characteristics of patients

A total of 220 patients were studied in Ireland and in Singapore. There were a total of 62 males and 43 females, 82 males and 28 females in Singapore during the May to July and October to December respectively. In Ireland, there were a total of 64 males and 46 females, 58 males and 52 females. The mean age, in years, in Singapore was 52.29 during the first period and 46.37 in latter, while in Ireland the mean age was 57.64 and 55.63. Table 1 and 2 provide a summary of the characteristics of the population in each country. The two populations involved in the study have relatively similar BMI. In Singapore, the BMI between the two periods are 28.9 and 26.9 while that in Ireland is 28.11 and 30.25.

### Seasonal difference between Ireland and Singapore

**Figure 1:**
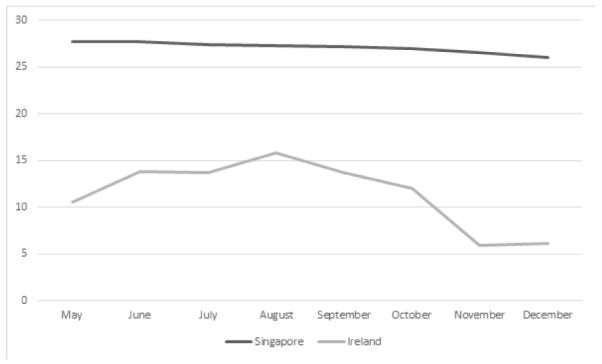
Seasonal variation in temperature in Ireland and Singapore (°C).

**Figure 2:**
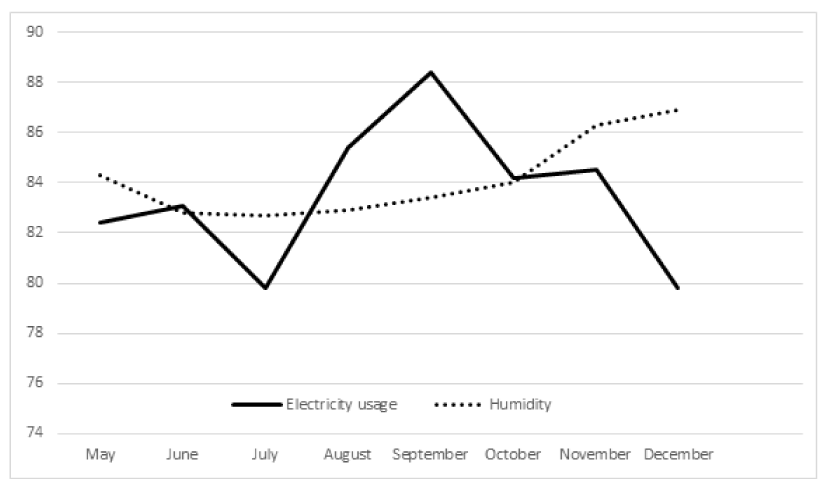
Changes in household usage of electricity (kWh) and humility (%) in Singapore.

**Figure 3:**
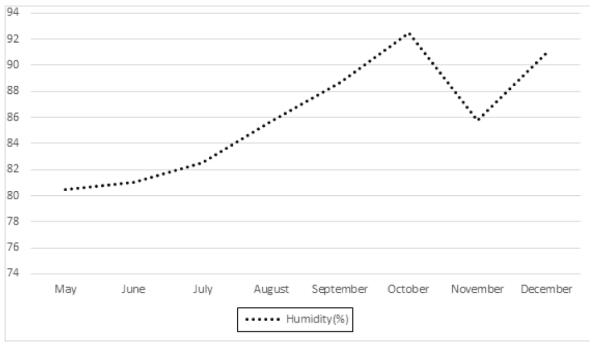
Changes in humidity (%) in Ireland.

Unlike Ireland, Singapore experiences a relatively stable temperature from May to December (Figure 1). This is because Singapore lies closer to the equator and does not experience any forms of seasonal changes. There is also a relatively stable humidity over these months (Figure 2). In contrast, Ireland experiences a significant rise in humidity over the periods from May to December (Figure 3). Singapore also does not experience any form of daylight shift as the number of daylight hours is generally 12 hours over the whole year, whereas Ireland experiences changes in daylight hours during different seasons. Changes in temperature, humidity and daylight hours all differed between Ireland and Singapore.

### Interactions between racial differences, temperature changes and blood pressure dipping

During the different seasons, there were changes in mean systolic and diastolic pressure dipping for both Ireland and Singapore as summarized in Table 3. The seasonal difference between Ireland and Singapore exhibited a significant change in diastolic dip of blood pressure (95% CI, 0.72 to 0.72, 6.03 mm Hg; p=0.013) as well as the duration of night time dipping in Ireland (95% CI, 0.045 to 1.01, 0.53 Hours; p=0.032) but not in Singapore. Surprisingly, systolic night time dipping in Singapore exhibited significant changes (95% CI, 0.38 to 4.83, p=0.022) whereas a non-significant trend was evident in Ireland (95% CI, 0.42 to 4.40, p=0.105). This could be linked to the greater usage of air-conditioning in Singapore and heating system in Ireland during the changes in temperature. This is associated with changes in electricity usage in Singapore in Figure 2. However, the relevant data of Ireland is not available.

When the average sleeping blood pressure between the two countries in the two seasons were compared (Figures 1 and 2), it was evident that Singapore did not experience as extensive a change in systolic BP (123.80 to 123.52 mm Hg), whereas diastolic BP increased instead, from 71.95 to 73.36 mm Hg). This is in contrast with the Irish average sleeping systolic BP which decreased from 119.14 to 117.21 mm Hg and diastolic which decreased from 65.82 to 62.55 mm Hg. This seems to show that despite mean systolic dipping changes in Singapore, the night time sleeping systolic and diastolic blood pressure hover around a constant value whereas that for Ireland changes due to the different seasons. The overall pulse pressure for Singapore decreased from 51.85 mm Hg to 50.16 mm Hg between summer and winter, while that for Ireland increased from 53.32 mm Hg to 54.66 mm Hg due to a greater decrease in diastolic pressure in Ireland.

**Figure 4:**
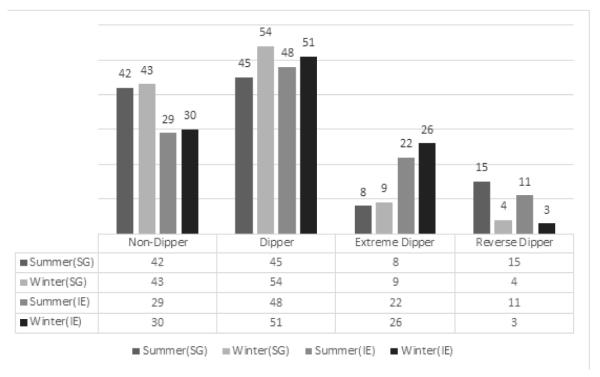
Number of different types of dippers during the summer and winter in Ireland and Singapore.

Despite the difference in temperatures and races, both the Irish and Singaporean populations showed relatively consistent changes in the type of dippers as seen in Figure 4. In both populations, there was a decrease in reverse dippers from the summer to winter period and an increase in number of dippers. In the Irish population however, there also seem to be an increase in the extreme dipper. This suggests that, regardless of the races or temperature, there seem to be a decline in total number of reverse dippers during the winter period as compared to summer. However, what differs in the two group is that, in the Asian population, the reverse dippers redistribute only to the dippers while in the White population, the reverse dippers redistributes to both the dippers and extreme dippers.

## Discussion

The results of this study show that there was not just seasonal link but also racial contrasts with the nocturnal dipping and the grouping in hypertensive patients. As seen in Table 4 there was a change in the diastolic dip in Ireland but not in Singapore. This is consistent with the findings of previous researchers who reported a link between seasonal changes in blood pressure due to increased vasodilatation during hot weather. It tends to affect the diastolic pressure more in hypertensive patients. It is estimated by Woodhouse et al. that a 1°C decrease in living room temperature will increase systolic and diastolic blood pressure by 1.3 mm Hg and 0.6 mm Hg respectively [17].

Figure 1 displays the seasonal change in temperature, which shows that the average change in temperature between summer and winter in Singapore and Ireland are 1.7°C and 7.8°C respectively. This would mean that the actual change of temperature in Ireland is 4.5 times that of Singapore. The change in mean dipping of diastolic blood pressure is also more significant in Ireland (3.4%) as compared to Singapore (2.1%). There was a trend towards a seasonal change in Singapore. This might attribute to the fact that, despite small, there was still a slight decrease in temperature in Singapore over the months from May to December, but may also reflect changes in humidity and possibly in the use of air conditioning. The value of mean dipping of diastolic blood pressure is an underestimation of the true value due to the low population size as well as changes in temperature in Cork during 2014 between the two seasons was ten degree Celsus (Figure 1), which may not be as extreme as many other countries, hence in more extreme temperature variations, the differences in blood pressure reading may be even bigger.

However, the systolic dip at night in Singapore was an unexpected change (p<0.05). In fact, the mean systolic dip changed a total of 2.6% as compared to 1.99% of Ireland. This could be due to many other variables which might have a greater effect on the systolic pressure. In the Irish population, there is an overall decrease in pulse pressure from 56.23 to 56.15 mm Hg while that in Singapore increased from 53.12 to 53.9 mm Hg.

In addition, the duration of night time dipping between the two seasons in Ireland changed from 2.99 to 3.52 hours, while that in Singapore was only from 2.57 to 2.97 hours. The result could also be an underestimation of the true value of the dipping as the average age in the Irish population is slightly higher. The average age for the Irish population is 58 and 56 between summer and winter while that in Singapore is 52 and 46 (Table 5). Age is one of the key factor which will increase the frequency of arousal from sleep [18], which might inevitably decrease the total duration of night time dipping in Ireland.

Interestingly, even though there are no changes in seasons in Singapore, both Ireland and Singapore seem to show an overall drop in the number of reverse dippers. The reverse dippers were being reattributed to only the dippers in Singapore while in Ireland to the dippers and non-dippers (Figure 4). This would suggest that the regardless of the temperature, reverse dippers tends to decrease during the year end while it is the racial differences which determines which group they would most likely be allocated to.

This study is unique in the inclusion of two countries, Ireland and Singapore, one of which has no seasonal changes. It allows the use of a single country to act as a form of control. Many present studies in similar field only use a single country as a form of comparison. This study thus, allowed us to further test the hypothesis.

### Limitations of the study

Some limitation of the study involves the fact that as data are being collected from different countries, the Ambulatory Blood Pressure Monitor (ABPM) being used is different which might give rise to systematic errors. However, both centers used systems of ABPM monitoring that are well validated and should minimize measurement bias in the collection of individual blood pressure values.

The strict selection on the population based on the exclusion and inclusion criteria as well as the small population of Cork city, resulted in a smaller than ideal population size. This also prevents the restriction of the age group, especially in Cork’s population. This is however offset by taking into account the confounding of arterial stiffness due to calcification with aging.

Both countries are very different in terms of culture, demographics as well as diet and that the Asian population tends to be lower in BMI as compared to the Caucasian population. Despite the differences in diet, people in Singapore are also affected by a wide range of westernization i.e. increase frequency of indulgence in many western diets such as fast food, high salt diet etc., thus frequently resulting in them having hypertension. Unlike Ireland, Singapore does not have winter season, thus the amount of food consumption are also generally more constant as people tend to consume greater amount and saltier food during the winter as compared to summer. This would inevitably affect the overall blood pressure as well.

However, despite the differences in diet, race and culture, the difference in BMI between both countries was modest. Furthermore, the results still showed similarities in the changes in the type of dippers between the two countries. This would suggest that the drop in reverse dipping during the winter season from summer was independent to any of the factors but purely due to the different periods of the year. The shift to other groups could, however, be affected by the different co-founders.

The population of hypertensive subjects studied in the summer and winter cohorts in each country differed. This is a reflection of the collection of this data from a real world population. Although it would be preferable to study the same population in both phases this was not practical in view of the clinical demand for ABPM and the need to apply it in an environment of routine care. As the comparison of an entire cohort in the two countries seems to show how the season will affect the dipping status in people, this would perhaps, be amplified if the same cohort were used.

Another limitation will stem from the fact that Singaporeans in general are mostly indoors in air-conditioned areas. During a hotter weather the air-conditioners will be used to reduce temperature. Irish on the other hand, will use heaters during the winter season and reduce the change in temperature due to any seasonal changes. In our population of non-night shift works the nocturnal BP recordings will almost exclusively be recorded in the patients’ homes. As a result, the changes in blood pressure might not accurately reflect the actual changes in the temperature changes due to different seasons as the air-conditioning and heating might lower the difference in changes of temperature due to the changes of weather. This might cause an underestimation of the actual change in blood pressure.

## Conclusion

In summary, it is difficult to obtain a single variable from two separate countries with different cultural influences. However, this research shows the pattern in changes of types of dippers in different races and the seasons. As reverse dippers are associated with a poor prognosis of cardiovascular diseases, clinicians have to pay attention to patients who might not present as a reverse dipper in non-winter seasons in particular patients from the dipper group in Singapore and both dipper and extreme dipper in Ireland. By taking the changes into consideration, clinicians will be able to control the changes in blood pressure more effectively in different races even when the seasons change through the control of dosages of medications prescribed. The change in blood pressure should also be taken into considerations when the patient travels into countries with different climatic environment.

## Figures and Tables

**Table 1:** Characteristics of subjects investigated in Singapore (n = 110, in each season).

Singapore	Range	Mean	Std. Deviation
Average age in summer (yrs)	17-90	52.29	20.57
Average age in winter (yrs)	16-89	46.37	19.18
Body Mass index in Summer (kg/m^2^)	16.8-47.8	28.9	24.3
Body Mass Index in Winter (kg/m^2^)	15.4-57.0	26.9	5.73
Average awake Systolic BP in Summer (mm Hg)	104.00- 167.00	135.36	14.03
Average awake Systolic BP in Winter (mm Hg)	109.00- 180.00	138.81	12.21
Average sleep Systolic BP in Summer (mmHg)	85.00- 168.00	123.8	16.4
Average sleep Systolic BP in Winter (mmHg)	97.00- 180.00	123.52	13.68
Average awake Diastolic BP in Summer (mm Hg)	46.00-117.00	80.4	11.31
Average awake Diastolic BP in Winter (mm Hg)	60.00- 121.00	83.59	11.47
Average sleep Diastolic BP in Summer (mm Hg)	50.00- 101.00	71.95	10.13
Average Sleep Diastolic BP in Winter (mmHg)	49.00- 120.00	73.36	10.56
Duration of night time dip in Summer (hours)	0-7.50	2.57	1.92
Duration of night time dip in Winter (hours)	0-7.50	2.99	1.77

**Table 2:** Characteristics of subjects investigated in Ireland (n = 110, in each season).

Ireland	Range	Mean	Std. Deviation
Average age in summer (yrs)	28-83	57.64	11.84
Average age in winter (yrs)	18-82	55.63	1434
Body Mass index in Summer (kg/m^2^)	15.0-49.6	28.11	4.97
Body Mass Index in VVinter (kg/m^2^)	18.5-42.3	30.25	5.43
Average awake Systolic BP in Summer (mm Hg)	105.00- 18330	135.71	1431
Average awake Systolic BP in Winter (mm Hg)	97.00- 17030	136.54	1337
Average sleep Systolic BP in Summer (mmHg)	87.00- 15930	119.14	14.93
Average sleep Systolic BP in Winter (mmHg)	78.00- 151.00	117.21	1329
Average awake Diastolic BP in Summer (mm Hg)	58.00- 10630	78.78	9.53
Average awake Diastolic BP in Winter (mm Hg)	56.00- 10230	79.17	9.97
Average sleep Diastolic BP in Summer (mm Hg)	47.00- 10030	65.82	9.73
Average Sleep Diastolic BP in Winter (mmHg)	45.00- 87.00	62.55	1126
Duration of night time dip in Summer (hours)	.00-7.50	2.99	1.92
Duration of night time dip in Winter (hours)	.00-8.00	3.52	1.74

**Table 3:** Mean systolic and diastolic dip between Singapore and Ireland during summer and winter.

Singapore				Ireland			
Mean systolic dip (%)		Mean diastolic dip (%)		Mean systolic dip (%)		Mean diastolic dip (%)	
Summer	Winter	Summer	Winter	Summer	Winter	Summer	Winter
8.34	10.94	9.9	12.01	11.93	13.92	16.06	19.44

**Table 4:** Comparison of type of dipper, systolic and diastolic dip, duration of night time dip between Singapore and Ireland.

	Mean	Levene Test for Equality of the Variance	Std. Error	95% Confidence Interval of the Difference		Significance
				Lower	upper	
Type of Dipper during Summer and Winter in Singapore	200	0.076	0.12	0.036	0.044	0.096
Type of Dipper during Summer and Winter in Ireland	0.118	0.054	0.12	0.11	0.35	0.309
Systolic dip at night during Summer and Winter in Singapore	2.61	0.005	1.13	38	4.83	0.022
Diastolic dip at night during Summer and Winter in Singapore	2.11	0.04	1.2	25	4.47	0.08
Systolic dip at night during Summer and Winter in Ireland	199	0.259	1.22	0.42	440	0.105
Diastolic dip at night during Summer and Winter in Ireland	337	0.814	1.35	0.719	6.027	0.013
Duration of night time dip between Summer and Winter in Singapore	0.42	0.28	0.25	0.073	0.91	0.095
Duration of night time dip between Summer and Winter in Ireland	0.53	0.199	0.25	0.045	1.01	0.032

**Table 5:** General demographics and coinorbidities of Irish and Singaporean population.

		Ireland		Singapore	
		Summer	Winter	Summer	Winter
Age	18-30	2	9	21	28
	31-50	30	29	29	35
	51-70	61	55	36	35
	71-90	17	17	24	12
Race	Chinese	-	-	96	99
	Malay	-	-	11	6
	Indian	-	-	3	5
	Irish	110	110	-	-
BMI	15-19^1^	4	1	9	5
	20-24^2^	20	13	41	41
	25-29^3^	52	55	38	42
	30-34^4^	26	24	13	13
	>35^5^	8	17	9	9

1 Underweight,

2 Normal

3 Overweight

4 Obese

5 Severly Obese
